# Strain Modulations as a Mechanism to Reduce Stress Relaxation in Laryngeal Tissues

**DOI:** 10.1371/journal.pone.0090762

**Published:** 2014-03-10

**Authors:** Eric J. Hunter, Thomas Siegmund, Roger W. Chan

**Affiliations:** 1 Communicative Sciences and Disorders, Michigan State University, East Lansing, Michigan, United States of America; 2 National Center for Voice and Speech, The University of Utah, Salt Lake City, Utah, United States of America; 3 School of Mechanical Engineering, Purdue University, West Lafayette, Indiana, United States of America; 4 Otolaryngology—Head and Neck Surgery, University of Texas Southwestern Medical Center, Dallas, Texas, United States of America; Northwestern University, United States of America

## Abstract

Vocal fold tissues in animal and human species undergo deformation processes at several types of loading rates: a slow strain involved in vocal fold posturing (on the order of 1 Hz or so), cyclic and faster posturing often found in speech tasks or vocal embellishment (1–10 Hz), and shear strain associated with vocal fold vibration during phonation (100 Hz and higher). Relevant to these deformation patterns are the viscous properties of laryngeal tissues, which exhibit non-linear stress relaxation and recovery. In the current study, a large strain time-dependent constitutive model of human vocal fold tissue is used to investigate effects of phonatory posturing cyclic strain in the range of 1 Hz to 10 Hz. Tissue data for two subjects are considered and used to contrast the potential effects of age. Results suggest that modulation frequency and extent (amplitude), as well as the amount of vocal fold overall strain, all affect the change in stress relaxation with modulation added. Generally, the vocal fold cover reduces the rate of relaxation while the opposite is true for the vocal ligament. Further, higher modulation frequencies appear to reduce the rate of relaxation, primarily affecting the ligament. The potential benefits of cyclic strain, often found in vibrato (around 5 Hz modulation) and intonational inflection, are discussed in terms of vocal effort and vocal pitch maintenance. Additionally, elderly tissue appears to not exhibit these benefits to modulation. The exacerbating effect such modulations may have on certain voice disorders, such as muscle tension dysphonia, are explored.

## Introduction

Flow-induced vocal fold oscillation is the basis of voice production in humans and most nonhuman mammals. Fundamental frequency (F_0_) is primarily changed through the stress within the vocal folds; and stress is a function of vocal fold stretch. Usually, the more a vocal fold becomes stretched (i.e., strain), the higher will be its oscillation rate and, therefore, F_0_. However, vocal folds are composed of tissues with complex time-dependent deformation characteristics. Once tissues are deformed by applying strain, the resulting stress is not constant, but instead decreases with time, a phenomenon known as stress relaxation. After vocal folds are shortened again and strain is released, the tissue recovers. Additionally, vocal fold tissues have a highly nonlinear stress-strain response in normal range of use [1].

Constitutive biomechanical models have been developed to describe these stress relaxation and recovery patterns in vocal fold tissues [2]–[4]. Several of these models have embodied the short-term cyclic strain effect in terms of mathematical models (e.g., two-network hyperelastic non-linear viscous model [5], Titze-modified Kelvin model [6]). However, such models only focused on characterizing the effects of short-term relaxation, which results in a banana like stress-strain curve with cyclic strain. Other models explore long-term stress relaxation and nonlinear effects [2]. More recently, Zhang et al. [1] reported on a model that combined these two effects by adding the nonlinear long-term relaxation effects to a two-network model of short-term relaxation, creating a three-network model with a hyperelastic response and nonlinear viscosity on two characteristic time scales. This model could theoretically account for the general effects of both the long-term step stress relaxation and short-term cyclic strain simultaneously. However, in Zhang et al. [1], only a proof of concept was shown in a simulation of a cyclic deformation history with step-wise changes in strain magnitudes—a situation not likely to occur in natural phonation.

**Figure 1:**
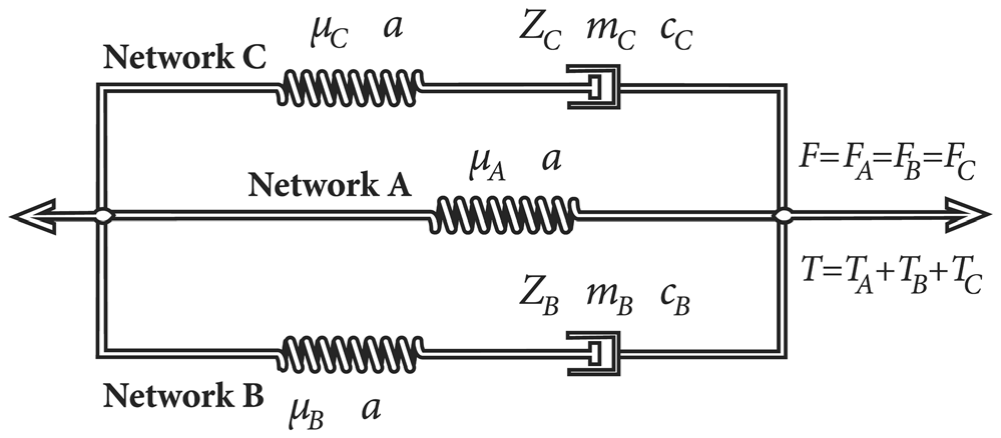
A rheological representation of the three-network constitutive model with a hyperelastic equilibrium network (A) in parallel with a short-term viscous network comp (B) and a long-term viscous network (C). Total tractions **T** are computed as the sum of the tractions in individual networks (**T** = **T_A_**+**T_B_**+**T_C_**) under the condition of equal deformation **F** in the individual networks (**F =  F_A_ =  F_B_  =  F_C_**).

The three-network model [1] is based on a rheological representation consisting of two hyperelastic equilibrium networks in parallel with two inelastic, time-dependent network components (Figure 1). It predicts laryngeal tissue stress when a dynamic strain is applied, and describes a strain energy density function as a power law of principal deformation components. The hyperelastic components of the model are based on the Ogden model [7], while those of the viscous terms relate to the formulation by Bergstrom and Boyce [8], [9]. The formulation of the model is explained in detail elsewhere [1], [10].

While no reference could be found in the literature, it is possible that small-amplitude recovery movements, i.e. small strain modulation, may change the rate of stress relaxation. This could be accomplished by redistributing elastic energy between the tissue components thus changing overall tissue relaxation. If this stress relaxation rate were changed by such strain modulations, these modulations could, in certain instances, provide a biomechanical advantage to vocal folds. Stress control in the vocal folds is the primary quantity in determining voice pitch. Therefore, there would be a stress relaxation effect on small-scale movements (strain modulations) which occur during such movements found in normal human vocalizations, such as running speech with voiced and unvoiced components at rates between 1 and 10 Hz during common speech tasks [11], [12] or vocal embellishments (e.g. vibrato, trillo, yodel) [13], [14]. They are probably also present in many mammalian calls, such as trill vocalizations [15]. The evidence of occurrence of vibrato and trill (strain modulated) during long vocalizations may imply the advantage of such vocalization types.

However, it has not yet been determined quantitatively if (and how) the effect of a short-term cyclic (modulated) strain superimposed onto a constant strain would change the overall long-term stress relaxation rate. Neither has it been determined how the related cyclic recovery would affect the overall stress in the tissue. In linear elastic systems, the overall average stress would not change when a constant strain with a smaller sinusoidal strain was applied. However, because the elastic component of vocal fold tissue is nonlinear with an exponential or power law type response [3], and because the viscous response is also nonlinear, a small sinusoidal strain superimposed on a constant strain may actually change the rate of stress relaxation.

The current study examined examples in human phonation of small vocal strain modulations on top of a large constant strain (e.g., tremor, vibrato, and trillo). Using a computer simulation, we hypothesized that there is an advantage to these types of modulation frequencies, which may enhance or mitigate general stress relaxation. If this hypothesis holds, relaxation would be larger if strain were kept constant and smaller if an additional small amplitude cyclic unloading and loading were superimposed on a large and constant strain. Simulations were accomplished using a three-network model [1]. Two cases were investigated. First, modulations with identical strain amplitude were varied between 0 Hz (no modulation) and 11 Hz. Second, modulation amplitude (or extent) was varied between 0 and 6% of the steady strain. For both cases, multiple instances of steady strain were simulated (5–60%).

## Materials and Methods

The methods of modeling the vocal fold tissue, including a discussion of the tissue parameters, was outlined first. Next, simulated experiments were described to test two strain modulation effects on stress relaxation.

### Three-Network Model

Vocal fold oscillation is critically dependent on the layered structure of vocal folds. The current study employs constitutive modeling developed previously but is presented here briefly for completeness [1] (Fig. 1, Table 1). The model consists of a time-independent equilibrium network based on the hyperelastic Ogden material model to give nonlinear elastic properties (Network A) and two viscoplastic networks to approximate time-dependent viscoelastic tissue short and long-term effects (two time scales). Network B captures the short-term, which would include the hysteretic response in cyclic loading, while Network C represents effects of stress relaxation and creep. This model, therefore, accounts for the general effects of the long-term constant strain (with stress relaxation) and short-term cyclic strain simultaneously.

**Table 1:** Model Parameters and a brief description of each.

Three-Network Ogden Model Parameters	
*Parameter*	*Description*
*µ_i_* (kPa)	Initial shear modulus for each network (*µ_A_*, *µ_B_*, *µ_C_*)
*Z_i_* (s^−1^ kPa^−*m*^ *_i_*)	Viscosity scaling constant for network B and C (*Z_B_*, *Z_C_*)
*m_i_*	Inelastic deformation dependence for B and C (*m_B_*, *m_C_*)
*c*	Stretch exponent (same for all networks)
*α*	Nonlinearity of elastic response (same for all networks)

### Model Tissue Parameters

The nine model parameters which characterize the biomechanical properties of human male vocal fold tissues were taken from previously published work [1]. To briefly summarize that study, tensile mechanical properties of tissue were measured using a sinusoidal stretch-release deformation of vocal fold tissue. Parameters of vocal fold cover and vocal fold ligament model from that study were chosen for two subjects to represent two distinctly different cases in which the viscous response is significant (33 y/o male) and suppressed (65 y/o male). In so doing, the two subjects represent a general trend in viscous response with age [16]. As presented in the previous work [1], tissue specimens were dissected from human larynges excised within 24 hours postmortem. Specimen preparation was done with instruments for phonomicrosugery. Each specimen was maintained at 37 C in Kregs-Ringer solution at pH 7.4 while mounted vertically to the lever arm (sutured) of a dual-mode server-control lever system (Aurora Scientific Model 300BLR, Aurora, ON).

### Modeling Tissue Response

The three-network model was implemented in MATLAB. Using the nine model parameters for both the younger and older tissue samples, the model could simulate the uniaxial stress response of the vocal fold cover and the vocal fold ligament to a uniaxial stretch. The response of the cover and ligament was simulated individually. Additionally, to approximate how the cover and ligament may respond together, a combined ligament and cover tissue was simulated. For the combined (quasi) stress response, the model was used to first calculate predicted stress for the cover and ligament individually for a single time step then the cover and ligament stresses were added (assuming a uniform and equal cross-sectional area of both). Thus, three virtual tissue specimen were modeled (*cov*: vocal fold cover; *lig*: vocal fold ligament; *tot*: combined cover and ligament) for a young and old case resulting in six total simulated tissue specimen.

**Figure 2:**
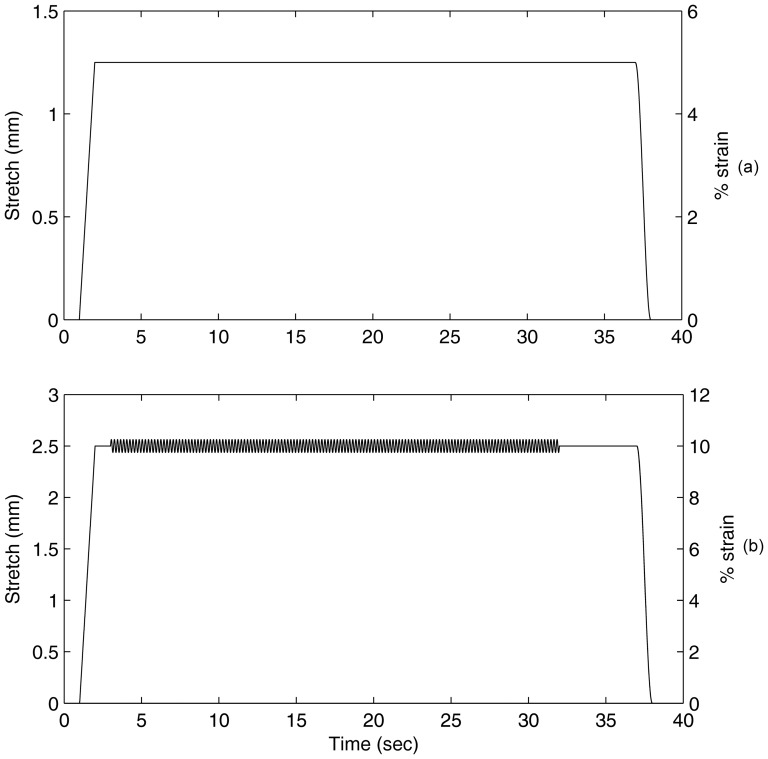
Simulated applied strain on laryngeal tissue model. (a) strain only (b) strain with modulation superimposed.

For each modeled tissue type (cov, lig, or tot), a modeled strain was applied as a single strain step held constant for 35 seconds (Figure 2a). This consisted of a 1.0 second rise-time and a 1.0 second fall-time on either side of the step period. Multiple steady strains between 5% and 60% of the tissue initial length were applied. This strain range is based on measurements during vocal performance. This scenario simulated a steady long-held phonation (>10 seconds), which was used as a baseline to compare two experimental modulations of the step strain.

### Simulated Experiments

Briefly, strain modulation was investigated through two experiments as described below. First, modulation frequency was varied to imitate a range of phonations with frequency modulations. Second, variations of modulation amplitude were added to a set modulation frequency.

### Effect of modulation frequency (Hz)

The first experiment investigated variations of modulation frequency. Strain modulation was added to the initial stepwise strain, starting after 1.0 second of constant strain and lasting for 35 seconds (Figure 2b). The modulation varied between 0 Hz (no modulation) and 11 Hz, resembling the range of vocal modulations for tremor, vibrato, and trillo. The strain amplitude of the modulation was 5% of the larger step strain (e.g., if the overall stepwise strain was 10% of the tissue length, modulation amplitude would be 5% of the 10% stepwise strain).

### Effect of modulation amplitude (% strain)

The second experiment investigated variations in modulation amplitude. Using a stepwise strain with a set modulation frequency of 6 Hz, the amplitude of the modulation was varied from 0 (no modulation) up to 6% of strain in terms of simulated F_0_ [17]. This maximum percentage was chosen based on classical operatic vibrato which has an extent (measured as the overall frequency range of the vibrato normalized to the sung pitch) of about 6% of the mean F_0_ [13]. Therefore, given a step strain, F_0_ was simulated [17] and a modulation amplitude to be added was calculated based on the percentage of the simulated F_0_.

The average decay rate of the stress-time response was obtained for each modulation input beginning from 1.0 seconds after the steady strain until 1.0 seconds before the strain release. The average decay rate was calculated as the difference in stress over the time interval of interest. While the dynamic stress change is not linear, the average decay rate was used as a basic approximation of the change in stress. The difference between the average decay rates of the predicted stress-time response for the modulated strain and the non-modulated strain condition was computed.

## Results

Results are divided into (1) variations of modulation frequency, and (2) variations of modulation amplitude (extent of modulation). Within these, an age-related case (younger vs. older tissue) is presented.

**Figure 3:**
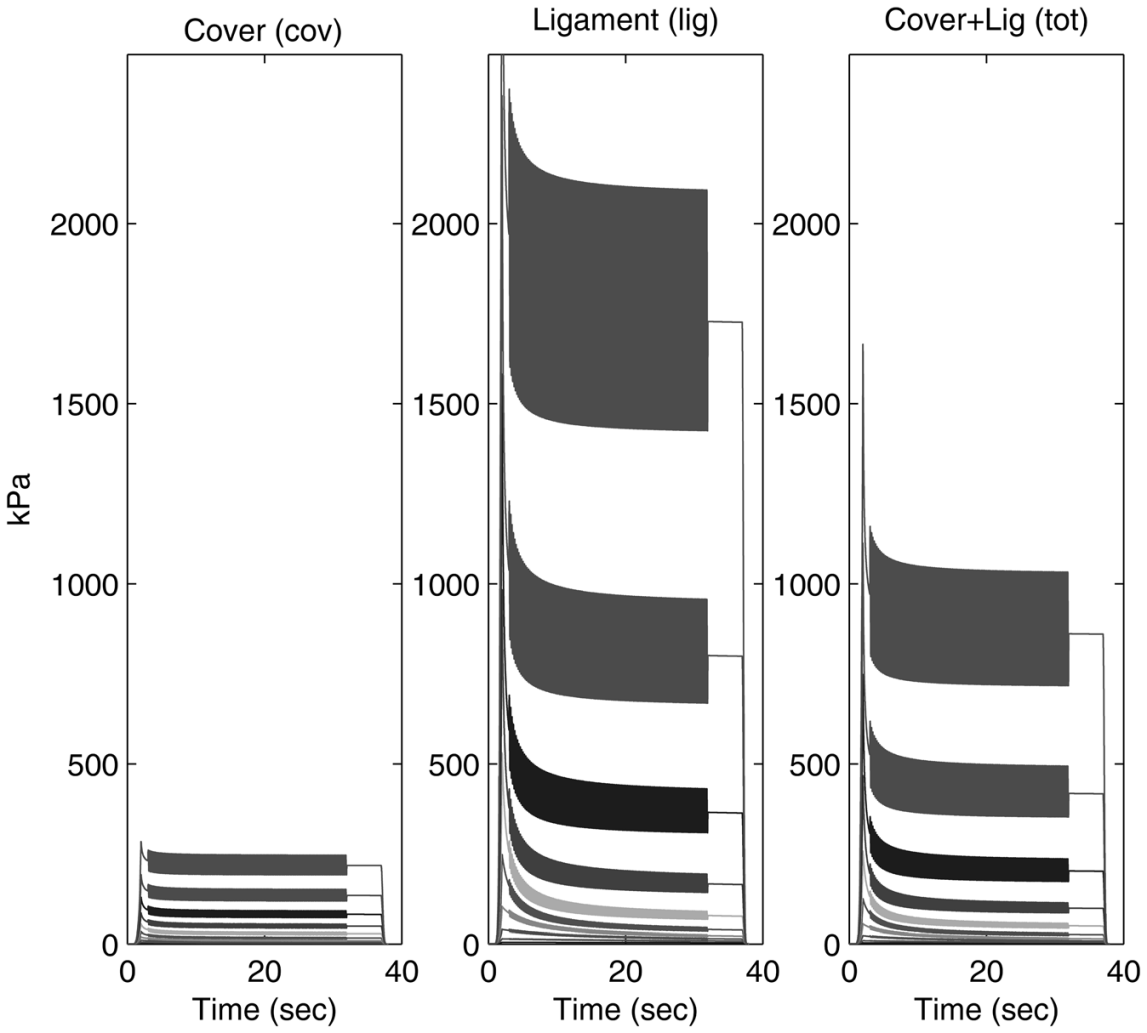
An illustrative result of simulated stress resulting from a modulated strain (e.g., Fig. 2b). Left: **cover** refers to the combination of epithelium and a superficial layer of lamina propria. Middle: **ligament** refers to the vocal ligament, which consists of the intermediate and deep layer of the lamina propria. Right: the cover and ligament responses summed together to suggest a combined tissue.

Actual vocal fold tissues relaxed over time and our simulated tissue captured this as shown in the simulated response of a single modulation frequency and a percentage extent but multiple overall strains (Fig. 3). In all cases, as is expected, the ligament demonstrated higher stress than the cover.

**Figure 4:**
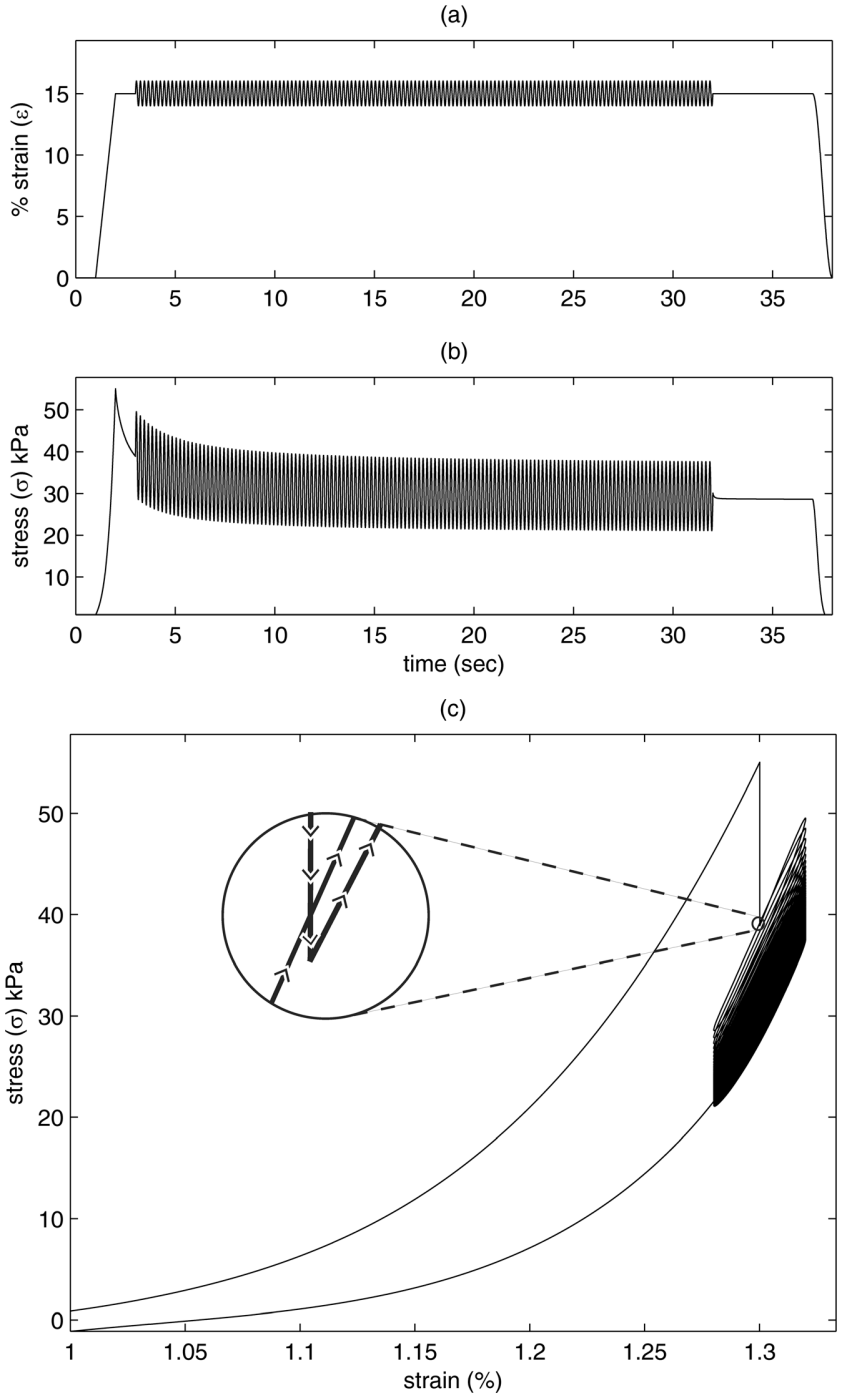
A specific simulated strain and stress response with a modulated strain: (a) strain input; (b) simulated stress; (c) strain plotted with stress. Highlighted portion (zoom in circle) shows part of the first cycle of modulated stress (arrows show direction in time) illustrating that the stress has slightly recovered and is above the original modulation cycle strain.

A specific case simulation is shown in Figure 4. For this case, stress peaked at 55 kPa for a strain of 30%. The tissue began to relax immediately thereafter (i.e., decreased stress). Strain modulation began 1.0 second after reaching peak strain. Before the onset of the small-amplitude oscillation, stress was at 40 kPa, i.e., 15 kPa had already been lost to viscousity. As modulation began, stress actually increased slightly by the end of the first cycle (Figure 4.c, zoom-in). For this particular strain and rate of cyclic modulation, mean stress during the second cycle was higher than during the first. Thus, there was some very small recovery associated with the strain modulation. This small recovery did not offset the overall stress lost during the hold period but reduced the amount of stress loss following the modulation onset.

Looking at the zoomed in portion of the first modulation cycle (in the highlighted circle), important details of the stress-stretch response is revealed. Within the zoom-in portion, stress decreases (nearly straight down following the arrows) then increases with a stretch (upward and towards the right direction). At the end of the first cycle of modulating stretch, the stress is higher than it was before the modulating stress begins (illustrated as the lower to upward and right line covering the full circle). As will be shown in further results, this small recovery is modulation frequency and modulation amplitude dependent.

The amount of short-term recovery depends on the magnitude of the initial strain amplitude, the strain modulation frequency (Hz) and the strain modulation amplitude (% strain). The difference between the average decay rate for constant strain and strain with modulation are illustrated as contour plots. A positive number indicates that the stress has been maintained with modulation better than with no modulation (i.e., decreased stress relaxation rate). In contrast, a negative number indicates that stress has been lost with modulation at a faster rate than with no modulation (i.e., increased stress relaxation rate).

### Variations in Modulation Frequency

**Figure 5:**
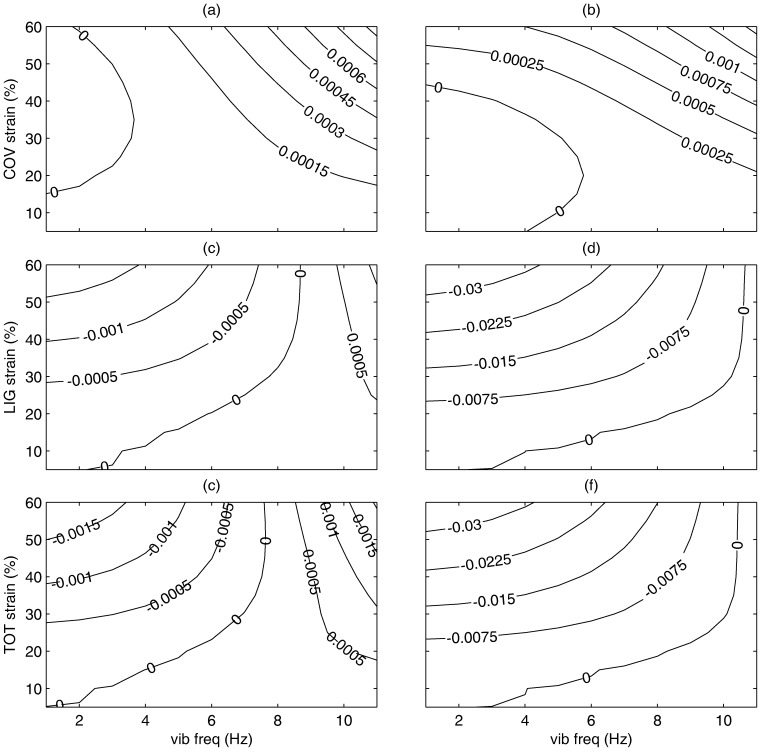
Simulated effect of modulation frequency and strain on stress relaxation for male laryngeal tissue model (top row- vocal fold cover; middle row- vocal ligament, bottom row- cover and ligament together). The left figures are for the 33/o male tissue model, the right figures are for the 66 y/o male model. Contour plots show the difference between the modulated strain case and no modulation with ‘0’ illustrating no difference. A positive number indicates that the modulation helped maintain the stress, whereas a negative number indicates a detrimental effect on stress (enhanced reduction). Modulation amplitude  = 5% of overall strain.

Thousands of simulations (similar to Figure 4) were conducted. From these, the difference between the average decay rates of the predicted stress-time response for the modulated strain and the non-modulated strain condition were computed. Figure 5, which shows the combined average differences in a contour plot as overall strain and modulation frequency changed. The plot illustrates the overall effect of strain modulation frequency on relaxation, demonstrating when the modulation helped maintain the overall stress (slowing stress relaxation, positive number) and when the modulation increased stress relaxation (negative number). Generally, for all simulated tissue the higher the modulation frequency was, the more stress was maintained and the less the tissue relaxed.

The simulated cover (Fig. 5a and b) resulted in the most stress maintenance (lack of relaxation) during the strain modulation trials; nowhere in the range of parameters investigated was the change of stress relaxation increased over the interval. This positive addition to stress (reduced stress relaxation) was not only greater at higher frequencies, but at higher overall strains. The younger tissue (Fig. 5a) had a net positive effect for modulation frequencies over 3 to 5 Hz when the tissue had some nominal strain (greater than about 10%). The older cover (Fig. 5b) responded generally similar to the younger tissue; however, both a higher modulation frequency and higher strain was needed to get this effect.

For the vocal ligament (Figure 5c and 5d), generally lower modulation frequency and higher overall strain resulted in an increased rate of stress relaxation. As the modulation frequency increased, the increase in rate of relaxation decreased until the rate of relaxation was the same as without modulation (occurring at about 8–8.5 Hz for the younger tissue specimen and at about 10–10.5 Hz for the older tissue specimen).

When simulating a combined cover and the ligament, (Figs. 5e, 5f), the ligament figures are adjusted slightly. Stress relaxation was more reduced when considering parameters characteristic of the younger of the two tissue specimens (Fig. 5e). For the younger specimen, the positive stress maintenance area (positive numbers) was larger than for the older specimen, with frequencies of no change in relaxation rate occurring near the range of normal sung vibrato frequency.

### Variations in Modulation Amplitude

**Figure 6:**
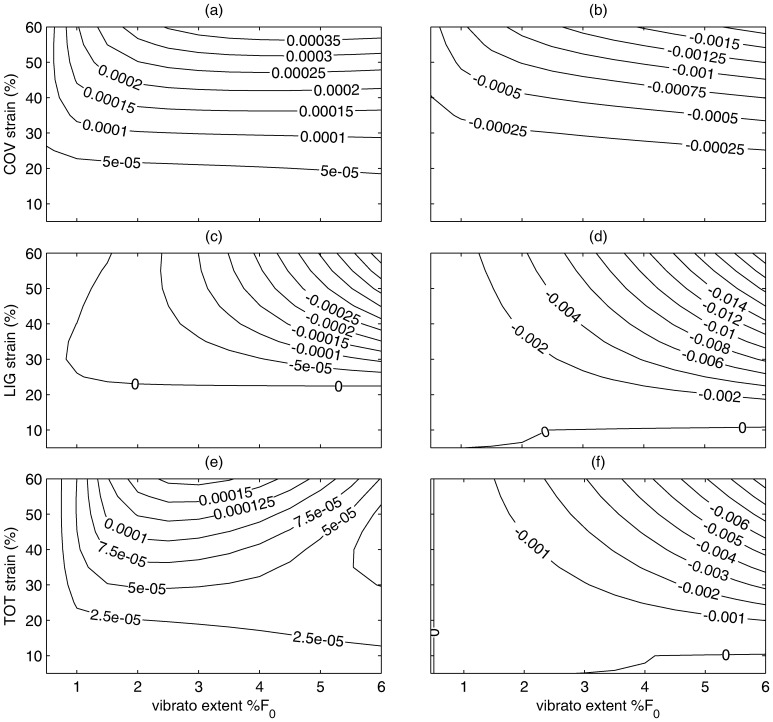
Simulated effect of modulation extent (modulation amplitude) on male laryngeal tissue model (top row- vocal fold cover; middle row- vocal ligament, bottom row- cover and ligament). Contour plots show the difference between the modulated case and no modulation with ‘0’ illustrating no difference. The 33 y/o male tissue is on the left; the 66 y/o male is on the right. A positive number indicates that the modulation helped maintain the stress where a negative number indicates a detrimental effect on stress. Modulation frequency  = 6 Hz.

Figure 6 illustrates the effect of modulation amplitudes. Modulation amplitude had little effect on relaxation of the vocal fold cover (Figs. 6a, 6b) for a modulation of 6 Hz with decreased stress relaxation rate for higher overall strains (positive maintenance). However, the rate of stress relaxation for the vocal ligament increased as the overall strain and amplitude of modulation increased, with higher amplitude of modulation and higher overall strain increasing the rate of relaxation.

The combination of cover and ligament illustrated a combined change of relaxation. For the younger specimen, the reduced stress relaxation of the cover is a larger effect than the increased relaxation of the ligament. The combined tissue (cover and ligament) for the younger tissue model had the greatest stress maintenance effect in the higher overall strains when combined with extents between 2–5% of the Fo. The model using the parameters from the older tissue resulted only in a negative effect or increased rate of relaxation in all three tissue cases (Fig. 6f).

## Discussion

The current study simulated the potential effects of small vocal strain modulations in a vocal fold tissue mode. Strain modulations are common in both singing and speaking, as well as in vocal disorders (e.g. essential tremor). They are also be seen in animal vocalization.

For the simulated younger vocal fold tissue, strain modulation of the vocal fold cover resulted in a reduced rate of relaxation over strain alone, while strain modulation of the vocal ligament resulted in increased rate of relaxation. Higher modulation frequencies reduced the rate of relaxation (better strain maintenance). Higher modulation amplitude (extent) seemed to primarily affect the ligament. There was also a difference in how the elderly and young tissue specimens responded to the modulation, with the elderly model resulting in mostly an increased stress relaxation effect because of the modulation.

From a mechanical standpoint, the observed effects could be understood from the interplay in the tissue response with respect to the mean strain and that of the modulation. Each modulation cycle could be separated into two phases with each phase possessing a stress relaxation response. First, the simulated stress overshot the equilibrium stress in the phase of increased strain, while the stress undershot the equilibrium stress during the strain release. Because the elastic responses of the tissue were nonlinear, as well as because the activation of the viscous response of the tissue was strongly dependent on stress, the stress overshoots seemed to be activated more strongly. If the modulation frequencies were low, the stress relaxation reached near equilibrium within each modulation cycle and no net effect was found. As the modulation frequency increased, the relaxation would be incomplete and a net effect of increased stress would remain. At given modulation conditions, the effect would be higher for a larger overall strain. If the modulation strain range was small, the relaxation process of the mean strain would dominate and modulation would not aid in stress maintenance.

For the situations where the difference in the results was positive (a reduction of the stress relaxation effect due to modulation), professional vocalists or occupational voice users who use their voice often may benefit. For example, classical singers who use vibrato (strain modulation) in the range of 5–6 Hz may be able to maintain the stress in the vocal folds longer, a useful advantage in high pitch notes held for several seconds. The results indicate that this would be an advantage in the vocal cover for almost any vibrato extent over the range studied. However, this advantage does not appear to extend to older singers (Fig. 6e, 6f), who may actually have a negative effect due to the vibrato. Per the results, an older singer would not want to use vibrato when singing higher frequency notes when there is significant strain since the vibrato would speed the relaxation of the tissue.

Modulation may be useful to frequent occupational speakers such as teachers or call center workers. It is possible that teachers' vocal effort could be reduced if the overall effort in maintaining voice were reduced (e.g., less lung pressure, less neck strap muscle involvement) by using increased intonation inflection in the voice.

However, the benefits of modulation might not be advantageous in some conditions. From the simulation, lower modulation frequencies did not help maintain stress. This would suggest that vocal conditions characterized by lower frequency modulation (e.g. 1–3 Hz), such as vocal tremors. In addition, higher strains would perhaps maintain a stress level that may in fact be detrimental. Thus, hyperfunctional voice disorders that are associated with presumably too high stress levels, such as Muscle Tension Dysphonia, could be exacerbated by modulation. Abductor spasmodic dysphonia also may be negatively affected by the modulations inherent to the disorder.

It is possible that these patterns are also observable for non-human mammals. For example, marmosets produce short, trill-like and non-modulated calls [15], [19], which are shorter than steady calls. It is possible that marmosets primarily use the vocal ligament (and at high modulation amplitude). Like humans, it is possible that the use of the high strain range at higher modulation amplitude and frequency may enhance stress relaxation (e.g., similar to Figure 6c and 6d), which may explain the shorter duration of these types of calls.

Presumably vocal fold tissue in vivo is continuously in a state of being used (strained) and not at rest. Therefore, there might not be the sharp initial decline of stress during frequent speaking as the (i.e., with the tissue in a quasi-steady state, with just a slight decrease in stress relaxation). So other than the initial startup of vocalization, when there has not been much straining of tissue for a few minutes, modulation might produce only a negligible effect in stress maintenance. Thus, one reason why vocal warm-up is necessary at the beginning of vocalization may be so that the stress of the system is more stable.

As this is a simulation study, there are some potential pitfalls. Because the modeling was based on parameters derived from the empirical data of two actual specimens (one old and one young), it is possible that these specimens were not representative of young and old laryngeal tissue. Nevertheless, the tissue samples of the young and old subject used seemed to be representative of other samples measured in Zhang et al. [16], and the differences in the results do correspond to general description of age dependent sustained phonations. Additionally, with the multiple tissues attached to other tissues in the larynx, the effects of modulated elongation on the tissues would never exist purely in isolation from which the model parameters were obtained. Therefore, the results from this simulation should be interpreted with the perspective that they represent only the specific specimen modeled and in these isolated cases.

In conclusion, the results of this study may have broader implications to occupational voice users such as teachers [18], who have a high instance of voice problems over most non-voice based occupations. Further, singers employing modulation techniques may be able to investigate slight modifications of their vibrato to find situations where a sung portion can be sustained longer than before. Finally, the effects on disorders with modulated like characteristics may actually be exacerbated by this effects. Nevertheless, we cannot predict the actual cumulative effect. Future modeling studies may use more extensive models of phonation [20] embedded with tissue models similar to those used in this report. This type of study would allow for better studies of the potential effect on the entire laryngeal system because we can only speculate how the communication mechanism would be affected given that the current findings are based on only the tissue constitutive model.

## References

[1] Zhang K, Siegmund T, Chan RW (2009) Modeling of the transient responses of the vocal fold lamina propria. J Mech Behav Biomed Mater 2: 93–104. 19122858 10.1016/j.jmbbm.2008.05.005 PMC2600447

[2] Alipour-Haghighi F, Titze IR (1985) Viscoelastic modeling of canine vocalis muscle in relaxation. J Acoust Soc Am 78: 1939–1943. 4078169 10.1121/1.392701

[3] Min YB, Titze IR, Alipour-Haghighi F (1995) Stress-strain response of the human vocal ligament. Ann Otol Rhinol Laryngol 104: 563–569. 7598370 10.1177/000348949510400711

[4] Perlman AL, Titze IR (1985) Measurement of viscoelastic properties in live tissue. In: Titze IR, Scherer RC, editors. Vocal fold physiology: biomechanics, acoustics and phonatory control. Denver, CO: The Denver Center for the Performing Arts.pp. 273–281.

[5] Zhang K, Siegmund T, Chan RW, Fu M (2009) Predictions of fundamental frequency changes during phonation based on a biomechanical model of the vocal fold lamina propria. J Voice 23: 277–282. 18191379 10.1016/j.jvoice.2007.09.010 PMC2742369

[6] Hunter EJ, Titze IR (2007) Refinements in modeling the passive properties of laryngeal soft tissue. J Appl Physiol 103: 206–219. 17412782 10.1152/japplphysiol.00892.2006

[7] Ogden RW (1972) Large deformation isotropic elasticity - on the correlation of theory and experiment for incompressible rubberlike solids. Proc R Soc London, Ser A 326: 565–584.

[8] Bergström JS, Boyce MC (1998) Constitutive modeling of the large strain time-dependent behavior of elastomers. Journal of the Mechanics and Physics of Solids 46: 931–954.

[9] Bergström JS, Boyce MC (2000) Large strain time-dependent behavior of filled elastomers. Mechanics of materials 32: 627–644.

[10] Hunter EJ, Palaparthi AK (2010) NCVS Memo No 12. Modeling Vocal Fold Tissue: Two- and Three-Network Ogden Models. http://www.ncvs.org/research_techbriefs.html. Accessed: 2014 Feb 13.

[11] Hunter EJ, Titze IR, Alipour F (2004) A three-dimensional model of vocal fold adduction/abduction. J Acoust Soc Am 115: 1747–1759. 15101653 10.1121/1.1652033 PMC1550351

[12] Titze IR, Hunter EJ, Svec JG (2007) Voicing and silence periods in daily and weekly vocalizations of teachers. J Acoust Soc Am 121: 469–478. 17297801 10.1121/1.2390676 PMC6371399

[13] Bretos J, Sundberg J (2003) Measurements of vibrato parameters in long sustained crescendo notes as sung by ten sopranos. J Voice 17: 343–352. 14513957 10.1067/s0892-1997(03)00006-7

[14] Titze IR, Solomon NP, Luschei ES, Hirano M (1994) Interference Between Normal Vibrato and Artificial Stimulation of Laryngeal Muscles at Near-Vibrato Rates. J Voice 8: 215–223. 7987423 10.1016/s0892-1997(05)80292-9

[15] Yamaguchi C, Izumi A, Nakamura K (2009) Temporal rules in vocal exchanges of phees and trills in common marmosets (Callithrix jacchus). Am J Primatol 71: 617–622 10.1002/ajp.20697 [doi] 19396872 10.1002/ajp.20697

[16] Zhang K, Siegmund T, Chan RW (2006) A constitutive model of the human vocal fold cover for fundamental frequency regulation. J Acoust Soc Am 119: 1050–1062. 16521767 10.1121/1.2159433

[17] Chan RW, Siegmund T, Zhang K (2009) Biomechanics of fundamental frequency regulation: Constitutive modeling of the vocal fold lamina propria. Logoped Phoniatr Vocol 1–9. 10.3109/14015430902913501 PMC2841710 19415568

[18] Hunter EJ, Titze IR (2010) Variations in intensity, fundamental frequency, and voicing for teachers in occupational versus nonoccupational settings. J Speech Lang Hear Res 53: 862–875. 20689046 10.1044/1092-4388(2009/09-0040) PMC3302664

[19] Tembrock G (1996) Akustische Kommunikation der Saugetiere. Wissenschaftliche Buchgesellschaft - WBG, Darmstadt.

[20] Titze IR, Hunter EJ (2007) A two-dimensional biomechanical model of vocal fold posturing. J Acoust Soc Am 121: 2254–2260. 17471739 10.1121/1.2697573 PMC6371396

